# Pragmatic application of a clinical prediction rule in primary care to identify patients with low back pain with a good prognosis following a brief spinal manipulation intervention

**DOI:** 10.1186/1471-2296-6-29

**Published:** 2005-07-14

**Authors:** Julie M Fritz, John D Childs, Timothy W Flynn

**Affiliations:** 1 Division of Physical Therapy, University of Utah, 520 Wakara Way, Salt Lake City, UT 84108, USA; 2 Rehab Agency, Intermountain Health Care, 2200 South 1685 West, Salt Lake City, UT, 84119, USA; 3 Department of Physical Therapy, Wilford Hall Medical Center, Lackland Air Force Base, San Antonio, TX, 78236, USA; 4 Department of Physical Therapy, Regis University, Denver, CO, 80221, USA

## Abstract

**Background:**

Patients with low back pain are frequently encountered in primary care. Although a specific diagnosis cannot be made for most patients, it is likely that sub-groups exist within the larger entity of nonspecific low back pain. One sub-group that has been identified is patients who respond rapidly to spinal manipulation. The purpose of this study was to examine the association between two factors (duration and distribution of symptoms) and prognosis following a spinal manipulation intervention.

**Methods:**

Data were taken from two previously published studies. Patients with low back pain underwent a standardized examination, including assessment of duration of the current symptoms in days, and the distal-most distribution of symptoms. Based on prior research, patients with symptoms of <16 days duration and no symptoms distal to the knee were considered to have a good prognosis following manipulation. All patients underwent up to two sessions of spinal manipulation treatment and a range of motion exercise. Oswestry disability scores were recorded before and after treatment. If ≥ 50% improvement on the Oswestry was achieved, the intervention was considered a success. Sensitivity, specificity, and positive likelihood ratio were calculated for the association of the two criteria with the outcome of the treatment.

**Results:**

141 patients (49% female, mean age = 35.5 (± 11.1) years) participated. Mean pre- and post-treatment Oswestry scores were 41.9 (± 10.9) and 24.1 (± 14.2) respectively. Sixty-three subjects (45%) had successful treatment outcomes. The sensitivity of the two criteria was 0.56 (95% CI: 0.43, 0.67), specificity was 0.92 (95% CI: 0.84, 0.96), and the positive likelihood ratio was 7.2 (95% CI: 3.2, 16.1).

**Conclusion:**

The results of this study demonstrate that two factors; symptom duration of less than 16 days, and no symptoms extending distal to the knee, were associated with a good outcome with spinal manipulation.

## Background

Low back pain (LBP) is a common and costly condition in primary care practices[1]. Managing patients with LBP is complicated by many factors, including the inability to identify a pathological cause for the majority of patients[2,3]. The fact that a specific diagnosis based on pathoanatomy can be made in only 10–20% of LBP sufferers seen in primary care leaves a large group of patients often given a nominal diagnosis such as "non-specific", or "mechanical" LBP.

Many experts agree that sub-groups exist within the large category of patients diagnosed with "non-specific" LBP[4,5]. The difficulty in identifying pathoanatomical causes in most patients combined with the high false positive rates of imaging studies[6] have led many to further conclude that meaningful sub-groups should be based on a patient's symptoms and clinical presentation [7-9]. The identification of sub-groups could improve the outcomes of clinical care by establishing more accurate prognoses, efficiently directing patients to therapies most likely to benefit their particular sub-group [10-12].

One proposed sub-group among patients with otherwise non-specific LBP consists of those who are likely to respond to spinal manipulation[13] There is evidence supporting the effectiveness of manipulation for patients with LBP,[14] however common sense, as well as research evidence,[15,16] recognizes that not all patients with LBP should be expected to respond to a manipulation intervention. The efficiency of primary care management of patients with LBP could be improved if a pragmatic tool existed to identify those patients with LBP who are likely to respond to this approach.

Flynn et al[16] examined factors from the clinical presentation of patients with LBP that predicted a successful response to two sessions of a manipulation intervention delivered by a physical therapist. Five clinical factors were identified as forming the most parsimonious set of predictors for identifying patients who achieved at least a 50% improvement in disability within one week with a maximum of two manipulation interventions (Table 1)[16]. The positive likelihood ratio for patients with at least 4 of these 5 criteria present was 24.4, which corresponded to a 95% likelihood of success with spinal manipulation among this sub-group of patients. Childs et al[17] sought to validate the effectiveness of these criteria for predicting a successful outcome from manipulation by randomizing patients to either a manipulation or strengthening/stabilization exercise intervention and examining outcomes based on the previously established criteria. The study found that patients with at least 4 out of 5 criteria present who received the manipulation intervention had better short-term (one- and four-week) and long-term (six-month) outcomes then patients receiving the manipulation who did not have at least 4 of 5 criteria. Furthermore, patients with at least 4 out of 5 criteria receiving manipulation had better short- and long-term outcomes than patients with 4 of 5 criteria who were randomized to receive the exercise intervention[17].

**Table 1:** Original criteria for predicting success with a manipulation intervention.17

**ORIGINAL CRITERIA – (at least 4 out of 5 must be present)**	
**Criterion**	**Definition of positive**

1. Duration of current episode of low back pain	< 16 days
2. Extent of distal symptoms	Not having symptoms distal to the knee
3. Fear-Avoidance Beliefs Questionnaire Work subscale score	< 19 points
4. Segmental mobility testing	At least one hypomobile segment in the lumbar spine
5. Hip internal rotation range of motion	At least one hip with > 35° of internal rotation range of motion

**PRAGMATIC CRITERIA – (both criteria must be present)**	

**Criterion**	**Definition of positive**

1. Duration of current episode of low back pain	< 16 days
2. Extent of distal symptoms	Not having symptoms distal to the knee

The results of these studies support the validity of these five criteria for identifying patients with LBP likely to benefit both immediately and in the long-term, from spinal manipulation. The pragmatism of applying these criteria for routine use in primary care, however, may be compromised by the requirements that the patient complete a questionnaire (the Fear Avoidance Beliefs Questionnaire[18]), and the clinician perform an examination of hip range of motion and spinal mobility. Therefore, the purpose of this report was to use data collected from previously published studies to examine the association between two pragmatic criteria (duration of symptoms and distribution of symptoms) and response to a manipulation intervention performed by a physical therapist.

## Methods

### Subjects

Subjects for this analysis were 141 patients with LBP who were participants in one of two previous studies. Seventy-one subjects were participants in the study used to develop the criteria for predicting success with manipulation,[16] and 70 subjects randomized to receive the manipulation intervention were taken from the study that validated the criteria[17]. These subjects underwent the same inclusion criteria, examination procedures, and treatment protocol and therefore were used to examine the validity of two criteria (duration of symptoms and distribution of symptoms) for predicting success with manipulation. Each study protocol was approved by the participating institutions and subjects all provided informed consent for participation.

Subjects were between 18–60 years of age with a primary complaint of LBP with or without referral into the lower extremity, and an Oswestry disability score of at least 30%. Patients with "red flags" for a serious spinal condition (e.g., tumor, compression fracture, infection, etc.), signs consistent with nerve root compression (i.e., positive straight leg raise <45°, or diminished reflexes, sensation, or lower extremity strength), current pregnancy, or prior surgery to the lumbar spine or buttock were excluded.

### Baseline examination

All subjects completed a series of self-report questionnaires and underwent a standardized physical examination prior to treatment. Subjects were asked to identify the date of onset of their LBP. Subjects also completed a body diagram to indicate the anatomical distribution of symptoms,[19] a numeric pain rating to indicate the current intensity of pain on a scale from 0–10[20], and the Fear Avoidance Beliefs Questionnaire to indicate the subject's fear of pain and beliefs about avoiding activity[18]. The modified Oswestry questionnaire (OSW)[21] was completed by each subject to quantify the level of disability related to LBP. The OSW is a well-validated tool for measuring disability in patients with LBP[22,23]. The version used in this study has been shown to possess high levels of reliability and responsiveness[24]. The OSW was completed at baseline, and again after one week of treatment.

### Treatment

**Figure 1:**
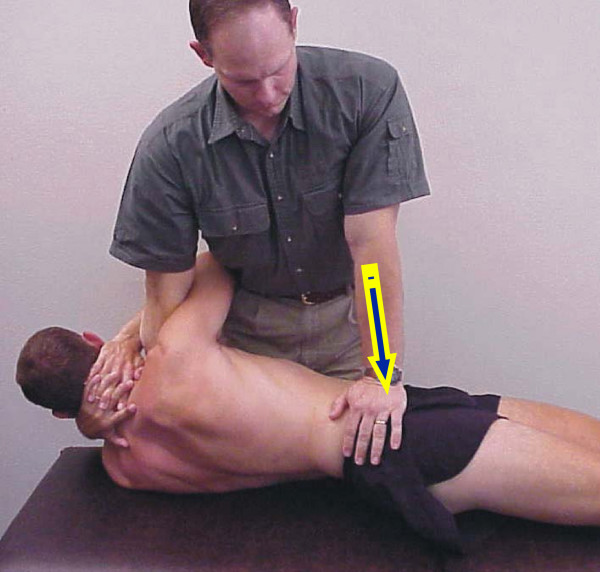
Manipulation technique.

After completing the baseline examination, all subjects received the same manipulation intervention protocol. Subjects were sent to physical therapy and received 1–2 treatment sessions within one week before re-examination. Subjects received the manipulation intervention at each treatment session. The same manipulation technique was used for all subjects. The subject was supine. The physical therapist positioned the subject into side-bending, and then rotated the subject in the opposite direction. A quick thrust to the pelvis was delivered by the therapist in a posterior and inferior direction (Figure 1). If a cavitation (i.e. a "pop") occurred, the physical therapist proceeded to instruct the patient in the range of motion (ROM) exercise. If no cavitation was produced, the manipulation was attempted again. A maximum of two attempts per side was permitted. The ROM exercise was performed supine by rocking the pelvis back and forth. Subjects were instructed to perform 10 repetitions of the ROM exercise in the clinic and 10 repetitions 3–4 times daily on the days they did not attend physical therapy.

### Data analysis

All subjects were categorized on two criteria from the baseline examination. The body diagram was used to determine if the subject's symptoms extended distal to the knee or not. The categorization of symptom distribution using body diagrams has been found to be highly reliable[25]. The subject's report was used to determine if the symptom duration was less than 16 days. Subjects with both criteria present (i.e., no symptoms distal to the knee *and *symptom duration less than 16 days) were categorized as likely to have a good prognosis following the manipulation intervention. Subjects with 1 or 0 criteria present were categorized as likely to have a poor prognosis.

We first examined the relationship between the two criteria rule and the original five-criteria rule for predicting success. Subjects with at least 4 out of 5 of the original criteria present were categorized as having a good prognosis with manipulation, while those with three or fewer were categorized as having a poor prognosis. Using the five-criteria rule as the reference standard, we calculated the accuracy of the two criteria as the percentage of times the two-criteria categorization agreed with the five-criteria categorization. We also calculated the sensitivity, specificity, and likelihood ratios with associated 95% confidence interval (CI).

The outcome of the manipulation intervention was determined from the change on the OSW occurring between the baseline and post-treatment examinations. The percent improvement on the OSW was calculated as (Initial _OSW _- Final _OSW_) /Initial _OSW_* 100%. If the percent improvement was ≥ 50%, the intervention was considered a success.

The association between the two criteria and manipulation outcome was examined by calculating the sensitivity, specificity, and positive likelihood ratio statistics with corresponding 95% CI. The sensitivity was calculated as the percentage of subjects with a successful outcome who had a good prognosis based on the presence of both criteria (i.e., the true positive rate). Specificity was calculated as the percentage of subjects with a non-successful outcome who were categorized with a poor prognosis based on the presence of only 1 or 0 criterion (i.e., the true negative rate). The positive likelihood ratio was calculated as (sensitivity / (1-specificity)) and represents the increase in odds favoring a successful outcome when both criteria are present[26].

## Results

The baseline characteristics of the 141 subjects are listed in table 2. At baseline, 105 subjects (74%) did not have symptoms distal to the knee, and 50 (36%) had a duration of symptoms < 16 days. Overall, 41 subjects (29%) had both criteria present and were categorized as having a good prognosis. One hundred subjects (71%) were categorized as having a poor prognosis; 73 had one, and 27 had zero criterion present. Characteristics of subjects with or without both criteria present are presented in table 3. Patients with both criteria present had higher baseline OSW scores (mean difference 5.6 points, 95% CI: 1.7, 9.5). The accuracy of the two criteria rule as compared with the original five criteria rule was high, with a percent agreement of 83.7%. The sensitivity and specificity were 0.67 and 0.93 respectively, resulting in a positive likelihood ratio of 8.9 and negative likelihood ratio of 0.36 (table 4).

**Table 2:** Baseline subject characteristics (n = 141).

**Characteristic**	
Age	35.5 (± 11.1) years
Sex	49% female
Duration of Symptoms	Median = 22 days (range: 1 – 2775 days)
Distribution of Symptoms	26% symptoms distal to the knee
Pain Intensity Rating	5.3 (± 2.0)
Fear-Avoidance Beliefs Questionnaire – Work Subscale	14.7 (± 10.3)
Oswestry Disability Questionnaire	41.9 (± 10.9)

(Numbers represent mean (± standard deviation) unless otherwise indicated)

**Table 3:** Baseline characteristics of subjects with or without both criteria for success with manipulation present

**Characteristic**	**Both criteria present (n = 41)**	**Both criteria not present (n = 100)**
Age	36.9 (± 11.4) years	35.2 (± 10.4) years
Sex	39% female	53% female
Pain Intensity Rating	5.6 (± 2.2)	5.1 (± 1.9)
Fear-Avoidance Beliefs Questionnaire – Work Subscale	14.0 (± 11.0)	15.0 (± 10.1)
Oswestry Disability Questionnaire*	45.9 (± 13.7)	40.3 (± 9.1)

(Numbers represent mean (± standard deviation) unless otherwise indicated, *significant difference between groups (p = 0.02))

**Table 4:** Accuracy of two criteria rule relative to the five criteria rule for predicting response to manipulation

	Likely responder*	Likely non-responder*
**Both criteria present**	32	7
**Both criteria not present**	16	86
	Sensitivity = 0.67 (95% CI: 0.53, 0.78)	Specificity = 0.93 (95% CI: 0.85, 0.96)
Positive Likelihood Ratio = 8.9 (95% CI: 4.2, 18.6)		
Negative Likelihood Ratio = 0.36 (95% CI: 0.24, 0.54)		

* The reference standard was the categorization of the subject based on the original five criteria prediction rule.

The mean baseline and one-week OSW scores were 41.9 (± 10.9) and 24.1 (± 14.2) respectively. The mean percent change on the OSW was 41.2% (± 33.9%). Sixty-three subjects (45%) experienced 50% or greater improvement on the OSW and were categorized as successful with the treatment (mean change 73.8% (± 15.4%)), and 55% were categorized as unsuccessful (mean change 14.8% (± 17.9%)). Subjects with both criteria present experienced significantly greater change on the OSW, and were more likely to be categorized as a successful manipulation outcome than subjects with one or zero criteria present (Table 5).

**Table 5:** Manipulation outcomes based on the number of criteria present

**Number of criteria present**	**Number of subjects**	**Number (%) with successful outcome**	**Percent change in oswestry (mean, (sd))**
0	27	2 (7.4%)	16.9% (23.4%)
1	73	26 (34.4%)	37.0% (32.7%)
2	41	35 (85.4%)	64.6% (27.2%)

The distribution of outcome with the manipulation intervention based on the presence of both criteria is displayed in table 6. The sensitivity associated with the presence of both criteria was 0.56 (95% CI: 0.43, 0.67), and the specificity was 0.93 (95% CI: 0.84, 0.96), with a positive likelihood ratio of 7.2 (95% CI: 3.2, 16.1).

**Table 6:** Accuracy of two criteria rule for success with manipulation for predicting clinical outcome

	Success*	Non-success*
**Both criteria present**	35	6
**Both criteria not present**	28	72
	Sensitivity = 0.56 (95% CI: 0.43, 0.67)	Specificity = 0.92 (95% CI: 0.84, 0.96)
Positive Likelihood Ratio = 7.2 (95% CI: 3.2, 16.1)		

*The reference standard was ≥50% reduction in Oswestry disability score.

## Discussion

The results of this study demonstrate a strong association between the two pragmatic criteria and response to a manipulation intervention delivered by a physical therapist. These two criteria (duration of symptoms less than 16 days and no symptoms extending distal to the knee) can be easily assessed in a primary care setting. The two pragmatic criteria also showed a high level of accuracy in relation to judgments based on the original five criteria (84% agreement, 67% sensitivity, 93% specificity). Judgments from the two criteria showed a strong predictive relationship with the clinical outcome. Although the two criteria rule did sacrifice some accuracy related to the original five criteria rule, it appears that sufficient accuracy is maintained with a substantial increase in ease of use. The original five criteria rule has been validated in a randomized clinical trial[17]. This two criteria rule will also require further validation through randomized trials to confirm that patients with both factors respond best to an intervention involving manipulation versus an alternative intervention, or no intervention at all.

Current clinical practice guidelines and expert recommendations generally support a "stepped care" approach for management of patients with LBP[27] in which treatment is initially limited to providing positive information and advice based on the understanding that the majority of patients will recover within 4–6 weeks. Referral to physical therapy may therefore not be initiated (ie, "stepped up") until patients fail to demonstrate the anticipated recovery. However, the results of this analysis, .along with our previous analyses,[16,17] suggest that patients with LBP seen in primary care whose duration of symptoms is less than 16 days without any symptoms extending distal to the knee, may benefit from an immediate referral to physical therapy for a couple of sessions of a manipulation intervention along with range of motion exercise. This analysis only examined results immediately after one week of treatment (up to 2 sessions), however the randomized trial by Childs et al[17] show that the benefit of early manipulation with range of motion exercise in patients fitting the criteria for success persisted up to six months after the treatment period. In addition, several studies have demonstrated that patients with LBP whose disability persists beyond 4–6 weeks are at significantly increased risk of developing chronic disability, persistent work restrictions, and increased health care utilization [28-31].

In this study, 29% of subjects fit the two criteria and were designated as having a good prognosis with manipulation, suggesting this sub-group of patients may not be inconsequential. The high specificity (0.92) and positive likelihood ratio (7.2) indicate that patients with both criteria present should be referred for a manipulation intervention based on the high likelihood of rapid success. The sensitivity (0.56) and negative likelihood ratio (0.48) associated with this two-criteria rule were only moderate, indicating a relatively high potential for false negative results (i.e., subjects designated as likely non-responders who ultimately experienced success with manipulation). Given the safety of manipulation in the lumbar spine[32], this finding suggests that referral of patients who do not have both criteria present may be appropriate in some cases. The criteria for treatment outcome with manipulation was determined after 1–2 treatment sessions, suggesting that the effectiveness of this treatment can be determined quickly in referred patients. Those patients who do not respond quickly may then be rapidly directed towards an alternative treatment approach. There is preliminary evidence to suggest that the addition of a strength and stabilization program after the manipulation intervention may help to reduce the likelihood of recurrence,[33] however more research is needed.

The exclusion criteria used in this study should be taken into account when considering the clinical implications of the results. In particular it is important to note that patients with signs of nerve root compression, low levels of disability, or prior surgery to the low back were excluded. The likely response of these patients to a manipulation intervention cannot be determined from these results. This study used one manipulation technique. It cannot be ascertained if the same criteria would apply to a different technique, however the lack of specificity associated with manipulation techniques[34,35] suggests that the choice of a particular technique may not be as important as the choice of the patient on whom spinal manipulation is to be used.

## Conclusion

Individuals with "non-specific" LBP are not a homogenous group, and different sub-groups of patients are likely to preferentially respond to different therapeutic management strategies. One sub-group consists of those patients with a good prognosis following spinal manipulation intervention. The results of this study demonstrate an association between two factors; symptom duration of less than 16 days, and no symptoms extending distal to the knee, and outcome of a manipulation intervention.

## Competing interests

The author(s) declare that they have no competing interests.

## Authors' contributions

JMF participated in the design and statistical analysis of this study. JDC participated in the design and performance of this study. TWF participated in the conceptual development and performance of this study.

## Pre-publication history

The pre-publication history for this paper can be accessed here:


